# Thematic Structure of Clinical Rehabilitation Research in the Early Post-Pandemic Period: An LDA-Based Analysis of Publications from 2022 to 2025

**DOI:** 10.3390/healthcare14162661

**Published:** 2026-08-21

**Authors:** Alina Alshevskaya, Ivan Khutornoy, Natalia Sivitskaya, Marat Fatkhullin, Elena Aksenova

**Affiliations:** 1 Federal State Autonomous Educational Institution of Higher Education I.M. Sechenov First Moscow State Medical University of the Ministry of Health of the Russian Federation (Sechenov University), 119991 Moscow, Russia; 2 State Budgetary Institution Research Institute for Healthcare Organization and Medical Management of Moscow Healthcare Department, 115184 Moscow, Russia; mcdm.avk@gmail.com (I.K.); sivitskayana@zdrav.mos.ru (N.S.); aksenovaei2@zdrav.mos.ru (E.A.); 3 Ivannikov Institute for System Programming of the Russian Academy of Sciences (ISP RAS), 109004 Moscow, Russia; marat779@gmail.com; 4 Moscow Institute of Physics and Technology, 141701 Moscow, Russia

**Keywords:** LDA topic modeling, medical rehabilitation, post-COVID-19 recovery, scientometric analysis, telemedicine, multidisciplinary approaches

## Abstract

**Highlights:**

**What are the main findings?**

- LDA-based analysis of more than 55,000 publications identified major emerging priorities in clinical rehabilitation, including telerehabilitation, virtual reality, wearable technologies, and psychosocial recovery.
- The proposed approach provided greater thematic coherence and more comprehensive coverage of interdisciplinary rehabilitation research than existing scientometric platforms.

**What are the implications of the main findings?**

- The identified trends can inform evidence-based planning of multidisciplinary, technology-assisted, and psychosocial rehabilitation services and research programs.
- Probabilistic topic modeling can support large-scale monitoring of scientific developments and strategic decision-making in rehabilitation research and public health policy.

**Abstract:**

**Background/Objective:** Recent advances in scientometric topic modeling have enhanced the capacity to rapidly assess evolving research priorities. This study aimed to characterize the thematic reorganization of clinical rehabilitation research during the early post-pandemic period and to compare how an Latent Dirichlet Allocation (LDA)-derived topic structure, SciVal, and OpenAlex represent this interdisciplinary research domain. **Methods:** Titles of 55,711 publications on clinical rehabilitation (2022–2025) were analyzed using an LDA-based topic modeling pipeline to identify predominant thematic directions. For comparison, topics automatically generated by Scopus and OpenAlex were examined. **Results:** The analysis revealed ten major thematic clusters encompassing the entire publication corpus. These clusters represented both medical and non-medical dimensions of rehabilitation research. The principal directions included post-COVID-19 recovery, psychological and quality-of-life outcomes, technological innovations such as virtual reality and telemedicine, and the growing emphasis on multidisciplinary and integrative rehabilitation frameworks. **Conclusions:** The proposed approach provided a field-wide, two-level representation of rehabilitation research and enabled a direct structural comparison of alternative topic-classification systems. The analysis showed systematic differences in aggregation, fragmentation, and corpus coverage across the compared approaches, without implying the universal superiority of any single method.

## 1. Introduction

The COVID-19 pandemic profoundly transformed the paradigm of medical rehabilitation, catalyzing a rapid shift toward remote and technology-assisted service delivery [1,2,3]. The necessity of maintaining continuity of care under pandemic restrictions accelerated the adoption of home-based and digitally supported rehabilitation [4,5]. Technological innovations, including telemedicine [6,7] and mobile health applications [8,9], became central to sustaining patient access to therapy. At the same time, the need for psychological support grew markedly, particularly in contexts of prolonged stress and social isolation [10,11]. Rehabilitation programs targeting post-COVID-19 recovery consequently emerged as integral components of clinical practice [12], emphasizing quality-of-life restoration and mitigation of asthenic symptoms across diverse patient populations [13,14].

**Figure 1:**
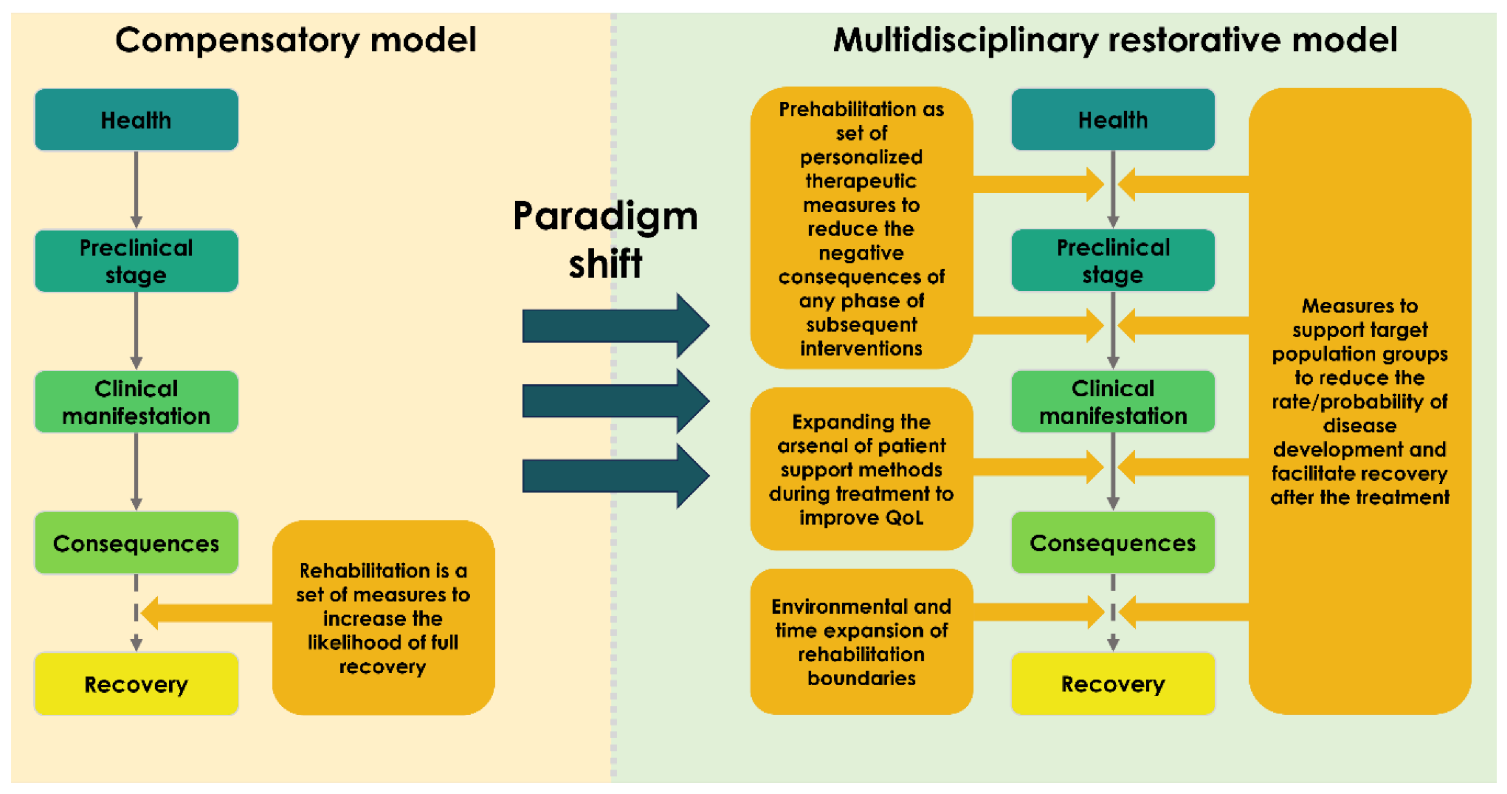
Paradigm Shift in Medical Rehabilitation from 2022 to 2025. Gray vertical arrows indicate the progression from health through the preclinical stage and clinical manifestation to consequences and recovery. Yellow horizontal arrows indicate the stages at which preventive, therapeutic, and rehabilitation measures may be applied. Dark teal arrows indicate the transition from the compensatory model to the multidisciplinary restorative model.

These developments underscored the importance of flexibility, adaptability, and innovation in rehabilitation systems to ensure continuous, patient-centered care during and after the pandemic. Consequently, rehabilitation practice began to move beyond the traditional framework of compensating for disease sequelae toward a comprehensive, multidisciplinary model that integrates prevention, restoration, and health preservation across all stages of medical care. Figure 1 provides a conceptual synthesis of changes described in the rehabilitation literature rather than a direct output of the subsequent topic-modeling analysis. In the compensatory model, rehabilitation is initiated primarily after the functional consequences of a disease or its treatment have become apparent, with recovery representing the principal endpoint. The multidisciplinary restorative model extends rehabilitation across the entire care pathway. It includes prehabilitation before therapeutic interventions, support aimed at maintaining function and quality of life during treatment, structured recovery after treatment, and longer-term measures addressing environmental and social barriers. At the clinical level, this model requires earlier identification of rehabilitation needs, shared goal setting, and coordination among medical, psychological, and social-care professionals. At the health-system level, it supports the development of connected rehabilitation pathways across inpatient, outpatient, home-based, and digitally supported settings.

This transformation also redefined the goals of rehabilitation. Rather than focusing exclusively on the mitigation of the most severe consequences of illness, the modern approach emphasizes maximizing recovery, social participation, and functional independence of the working-age population [15]. The focus has thus evolved from a compensatory to an active, restorative paradigm, shifting attention from disability toward the optimization of individual potential and capabilities [16,17].

The new paradigm also highlights the importance of adequate support and resources for recovery and long-term well-being. This support encompasses accessible medical services [18,19], physical rehabilitation [20], and psychological rehabilitation [21]. It also requires a social environment that promotes inclusion and independent living [22]. Evidence indicates that integrated rehabilitation and social-support programs not only enhance quality of life but also improve long-term clinical outcomes [23].

Despite these advances, an unresolved challenge concerns the identification of specific research and technological frontiers within rehabilitation medicine. Recognizing current scientific trends enables clinicians and policymakers to incorporate emerging technologies and therapeutic concepts, thereby improving patient outcomes [24]. Systematic monitoring of these trends is essential for adapting to evolving patient needs, implementing personalized interventions, and integrating innovative approaches into clinical practice to enhance quality of life.

A growing number of data-driven technologies, collectively termed topic modeling, are now used to explore emerging scientific frontiers. Some of these approaches are integrated into major bibliometric platforms such as Scopus and Web of Science, while others, including OpenAlex, are developed on open datasets and remain in active evolution. Preliminary analyses of post-COVID-19 rehabilitation research across these systems indicate methodological limitations, including fragmented topic structures and limited semantic coherence. To address these challenges, we applied a Latent Dirichlet Allocation (LDA)-based approach, one of the most established probabilistic topic-modeling methods [25,26], to obtain a more integrated mapping of research trends. Previous applications of LDA in rehabilitation research have primarily focused on narrowly defined domains, such as telerehabilitation for cerebral palsy and virtual-reality-based motor and balance rehabilitation. In contrast, the present study examines clinical rehabilitation as a whole, providing a corpus-wide analysis of its thematic structure during the early post-pandemic transition. The proposed framework combines field-level topic modeling with two-level topic refinement, expert-assisted semantic validation, and a structural comparison with SciVal and OpenAlex, enabling a comprehensive assessment of how the organization of rehabilitation research has evolved across the entire domain. Accordingly, the objective of this study was to identify and characterize current thematic directions in clinical rehabilitation during the post-COVID-19 period and to delineate emerging scientific and technological frontiers in this field.

Accordingly, the objective of this study was to map and characterize the thematic structure of clinical rehabilitation research during the early post-pandemic period. In addition, we examined how different topic-formation approaches differ in their representation of thematic aggregation, coverage, and internal organization within this research domain.

## 2. Materials and Methods

### 2.1. Study Design

This study employed a structured, multi-stage analytical workflow designed to ensure reproducibility and methodological transparency. LDA model was used for probabilistic topic extraction and clustering of publications related to clinical rehabilitation. All data pre-processing and model implementation were performed in Python 3.10, which provided a unified environment for corpus management, text cleaning, and computational modeling.

### 2.2. Data Retrieval

**Figure 2:**
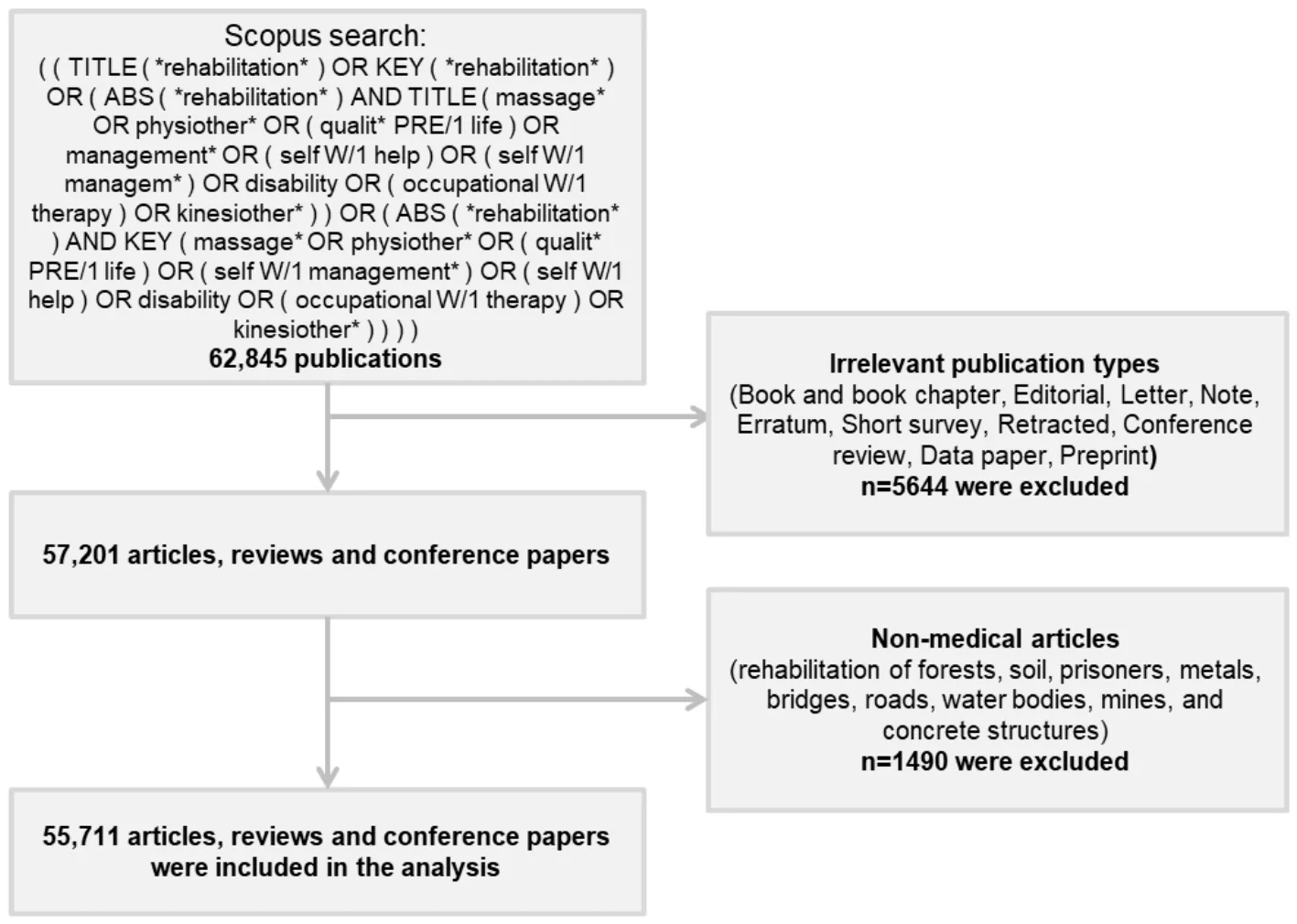
Flowchart of article selection for analysis (last update—25 March 2025).

Bibliographic records were retrieved from the Scopus-indexed literature through the SciVal platform. The search covered publications issued between 1 January 2022 and 25 March 2025. The approximately three-year analytical window from 2022 to early 2025 was defined a priori to capture the early post-pandemic transition in clinical rehabilitation research, when COVID-19-related effects remained prominent while broader research priorities were being reorganized after the acute pandemic phase. Potentially relevant records were identified among English-language publications. Eligible records contained the term “rehabilitation” in the publication title or in the author or indexed keywords. Alternatively, “rehabilitation” could occur in the abstract together with at least one of the following terms: “physiotherapy,” “massage,” “quality of life,” “management,” “self-help,” “disability,” “occupational therapy,” or “kinesiotherapy.” The complete search expression is provided in Figure 2.

The initial search returned 57,201 records. The exported dataset included publication title, abstract, author keywords, indexed keywords, publication year, document type, source title, DOI, citation count, and other fields not utilized in this study. Only articles and reviews published in English were retained.

Potentially non-relevant uses of the term “rehabilitation” outside a clinical or healthcare context were identified based on publication titles and, where necessary, abstracts and keywords. Three reviewers independently identified and verified the non-clinical terms and categories used as exclusion criteria, with disagreements resolved through discussion with an additional reviewer. These categories included the rehabilitation of forests, soils, prisons, metals, bridges, roads, water bodies, mines, and concrete structures, as summarized in Figure 2. Application of the agreed exclusion criteria resulted in the removal of 1490 non-relevant records and a final dataset of 55,711 publications (Figure 2).

The unit of analysis for topic modeling was the individual publication title. Abstracts and keywords were used only during record retrieval and relevance screening and were not included in the final LDA corpus. Consequently, the analytical dataset consisted of 55,711 title-level text documents accompanied by their corresponding bibliographic metadata.

### 2.3. Data Processing

The initial dataset, exported from SciVal, underwent standardized natural-language preprocessing. Tokenization was performed using the SpaCy library version 3.8.7 [27] with the en_core_web_sm 3.8.0 model. The tokenizer was modified to treat hyphenated words as single tokens, preserving domain-specific terms (e.g., post-stroke, long-COVID). A customized cleaning pipeline removed potential sources of bias, including numeric values, special characters, punctuation, adverbs, pronouns, and semantically insignificant stopwords (e.g., systematic, review, significant). Lemmatization was then applied to normalize inflected forms into their base lemmas, reducing lexical variability. Rare and overly frequent tokens were filtered out to enhance topic precision: words appearing in fewer than 25 articles or in more than 50% of articles were discarded. For sub-topic modeling, an additional threshold removed tokens occurring in more than 30% of documents to yield finer thematic granularity.

Model validation followed a multidimensional strategy combining quantitative, qualitative, and comparative criteria. Because LDA is an unsupervised method and no predefined reference taxonomy or ground-truth topic labels were available for the analyzed corpus, validation was not based on classification accuracy. Internal model quality was evaluated using the c_v coherence score, topic diversity, and log perplexity. These metrics were interpreted jointly: coherence assessed the semantic relatedness of the most representative terms within each topic, topic diversity measured redundancy among the top-ranked words across topics, and log perplexity characterized the probabilistic fit of the model to the corpus. Semantic interpretability was additionally evaluated through independent expert verification and refinement of the generated topic labels. Finally, the resulting topic structure was externally contextualized through comparison with SciVal and OpenAlex in terms of corpus coverage, thematic fragmentation, and interpretability. No single metric was treated as conclusive; the final topic configuration was selected based on the combined evidence provided by these validation components.

### 2.4. Generating Topics and Evaluating Model Performance

LDA is a generative probabilistic model. Each document is represented as a mixture of latent topics, and each topic is characterized by a probability distribution over words. For document d, represented in this study by an individual publication title, the topic distribution is sampled as θd∼Dir(α). For latent topic k, the word distribution is sampled as ϕk∼Dir(β). At each word position n, a topic assignment zd,n is drawn from θd. The observed word wd,n is then generated from the corresponding topic–word distribution ϕzd,n. The hyperparameters α and β control document–topic and topic–word sparsity, respectively.

This probabilistic mixed-membership structure enables a publication to contribute to more than one latent theme and supports unsupervised, corpus-wide mapping without predefined classification labels [24]. It is particularly useful for the exploratory identification of broad thematic domains and their subsequent refinement into more specific subtopics. Among the available approaches, LDA was selected because it provides transparent document–topic and topic–word probability distributions, supports mixed thematic membership, and can be applied consistently to a large short-text corpus. This choice was based on interpretability and computational feasibility rather than an assumption of general superiority over more recent contextual or neural topic-modeling methods. However, LDA uses a bag-of-words representation and therefore does not directly account for word order, contextual meaning, or semantic equivalence. Its outputs may also vary with preprocessing choices, the selected number of topics, hyperparameter settings, and random initialization, while topic interpretation and labeling require expert judgment. Accordingly, LDA was used here as an exploratory mapping tool rather than as a definitive classification or predictive model.

LDA training was implemented using Gensim version 4.3.3 [28]. Pre-processed tokens were vectorized and filtered according to the thresholds above to optimize topic separation. The optimal number of topics was determined empirically by examining the coherence score (c_v) curve, selecting the value that maximized semantic consistency among top-ranked terms. The LDA hyperparameters were specified a priori as α = 0.01 for the document–topic distribution and β = 0.9 for the topic–word distribution. The low α value favors sparse document–topic distributions, which was considered appropriate for the present short-text corpus because an individual publication title is generally expected to contain only a limited number of dominant themes. The comparatively high β value provides greater smoothing of the topic–word distributions, allowing topics to be represented by a broader set of semantically related terms rather than by only a few highly dominant words. These parameters were maintained consistently across all primary and sub-topic models to support comparability of the resulting topic structures and were not intended to represent an empirically optimized configuration. Since the present study was designed as an exploratory bibliometric and semantic mapping analysis, additional hyperparameter sensitivity testing was not performed. Model quality was assessed using standard evaluation metrics—coherence, topic diversity, and perplexity—to confirm the robustness and interpretability of the final topic configuration. Topic diversity was calculated as the proportion of unique words among the top words across all topics, with higher values indicating lower redundancy and better differentiation between topics. Perplexity was used to evaluate the probabilistic fit of the model to the corpus, with lower values indicating better model generalization.

### 2.5. Topic Naming and Relative Weight

Each topic was initially assigned a provisional descriptive label using a transformer-based generative language model based on the ten highest-probability terms. Two authors with domain expertise then independently evaluated the semantic consistency and clinical relevance of each topic using its highest-probability terms and representative publications with high posterior topic probabilities. The provisional labels were retained or refined through expert consensus. This procedure constituted topic-level semantic validation and did not involve manual screening or data extraction from every publication in the corpus. Relative topic weights were computed as the proportion of corpus documents assigned the highest posterior probability for a given topic.

### 2.6. Visualization

Visualization of topic relationships and keyword distributions was conducted with the pyLDAvis package version 3.4.1 [29] using default interactive parameters. This tool provided a two-dimensional representation of inter-topic distances and token frequencies, facilitating intuitive exploration of thematic structures and overlaps within the dataset.

## 3. Results

### 3.1. Determination of the Optimal Number of Topics

**Figure 3:**
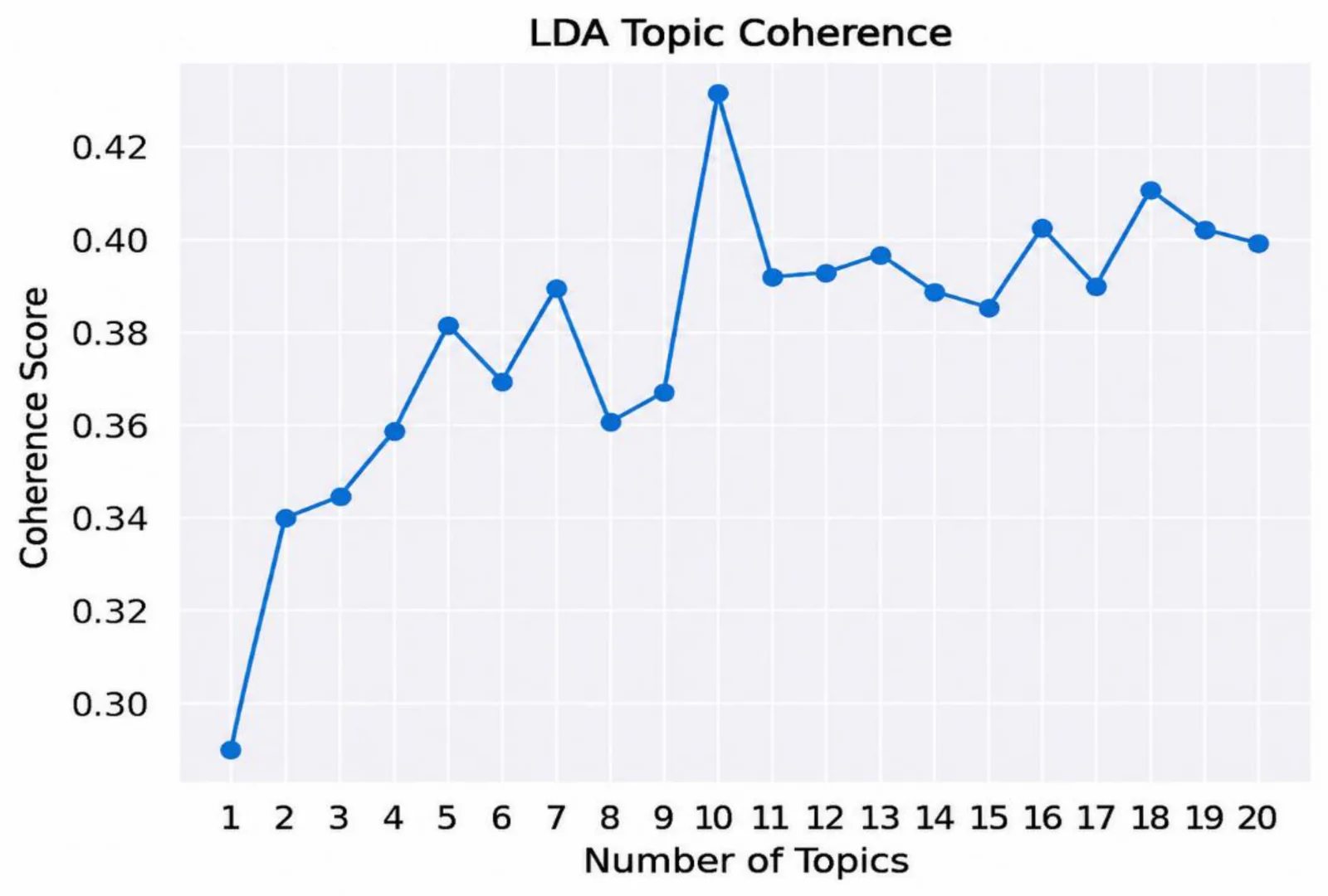
Selection of the optimal number of thematic clusters. The coherence (C_v) value for 10 topics = 0.43.

Primary modeling indicated that the ten-topic configuration provided the most suitable balance between thematic resolution and interpretability among the evaluated solutions (Figure 3). For this configuration, the c_v coherence score was 0.43, topic diversity was 0.86, and log perplexity was −8.31. The relatively high topic-diversity value indicated limited repetition of the most representative terms across topics, while the coherence score supported the semantic interpretability of the extracted term groups. Log perplexity was considered as a complementary measure of probabilistic model fit rather than as an independent selection criterion. The quantitative indicators were interpreted together with expert assessment of the topic-word distributions and the meaningfulness of the resulting thematic labels. The ten-topic solution was therefore retained because it provided an interpretable and sufficiently differentiated representation of the corpus rather than because of any single metric alone.

### 3.2. Thematic Structure of Rehabilitation Research

**Figure 4:**
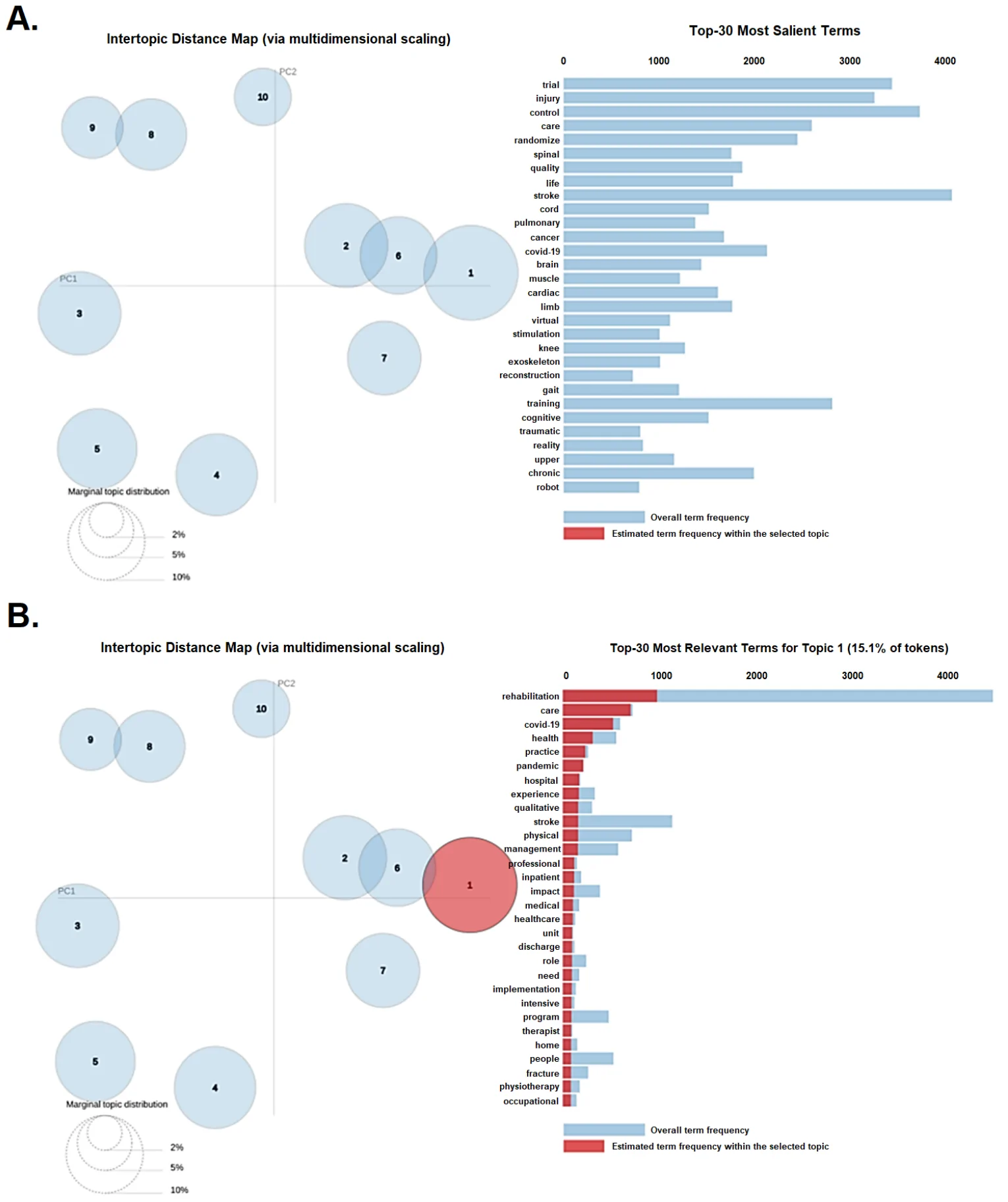
Visualization of thematic groups and key tokens in 55,711 rehabilitation-related articles (**A**) and example of token distribution within a topic (**B**). Each circle represents one LDA-derived topic, and circle size reflects its relative prevalence in the corpus. The distance between circles indicates semantic proximity between topics based on topic–word distributions in the reduced two-dimensional topic space. Partial overlap of circles reflects close semantic proximity and shared title-level vocabulary between the corresponding topics. Quantitative topic-level analyses were performed using dominant-topic assignment of individual publications. Saliency of terms is calculated based on the following formula: $saliency\left(w\right)=P(w)\times \sum_{T}P\left(T|w\right)log\frac{P(T|w)}{P(T)}$, where w stands for word, $P\left(T|w\right)$ is the likelihood that observed word w was generated by latent topic T and P(T) is the likelihood that any randomly selected word was generated by topic T [30].

The ten clusters identified through LDA modeling encompassed the full spectrum of research directions in clinical rehabilitation from 2022 to 2025 (Figure 4). Each topic was characterized by its representative terms and dominant subthemes.

The following ten thematic domains were identified:

1. COVID-19 and Rehabilitation Care: Health outcomes, quality of life, and patient-reported experiences in post-COVID-19 recovery (n = 5390);
2. Cancer and Cardiac Rehabilitation: Integration of exercise programs, perioperative management, and cardiovascular interventions (n = 9140);
3. Knee Rehabilitation and Robotics: Exoskeleton-assisted and robot-supported therapy after arthroplasty (n = 6080);
4. Traumatic Brain Injury and COVID-19 Cohorts: The role of virtual reality in neurological and cognitive rehabilitation (n = 2230);
5. Stroke Rehabilitation: Gait and limb stimulation, upper-limb training, and mobility restoration (n = 5387);
6. Childhood Disability and Spinal Cord Injury: Pediatric rehabilitation and developmental assessment (n = 5894);
7. Evaluation of Rehabilitation Protocols: Meta-analyses of randomized trials and pain management interventions (n = 8461);
8. Pulmonary and Cardiac Comorbidity Rehabilitation: Chronic obstructive and heart failure rehabilitation programs (n = 1511);
9. Muscle Strength and Ligament Reconstruction: Functional recovery after anterior cruciate ligament repair (n = 7404);
10. Fracture Management and Orthopedic Recovery: Surgical outcomes and rehabilitation following lumbar and cervical fractures (n = 4214).

Collectively, these clusters demonstrate that modern rehabilitation research spans a broad range of medical, technological, and psychosocial dimensions, with a marked emphasis on technology-assisted and patient-centered approaches.

### 3.3. Sub-Topic Modeling and Intra-Topic Structure

To further refine thematic interpretation, sub-topic modeling was conducted within each primary cluster. This process revealed distinct micro-level research focuses, providing a higher-resolution view of thematic heterogeneity.

Each sub-topic was defined by its unique keyword constellation and thematic orientation, often reflecting specialized domains within the parent topic—for example, differentiating robot-assisted knee therapy from tele-supervised physiotherapy within the same overarching cluster.

**Figure 5:**
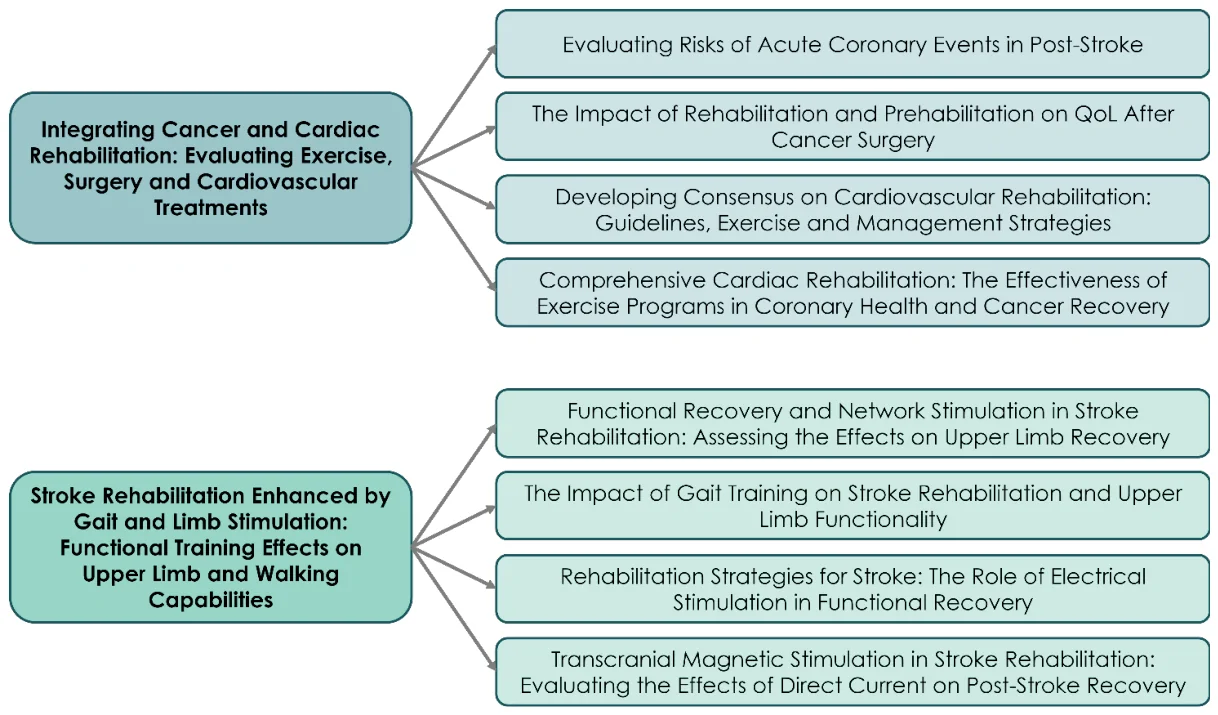
Sub-topics examples for primary topics 2 (**on the top**) and 5 (**on the bottom**).

Such sub-topic analyses enabled detection of emerging research niches, including remote rehabilitation protocols, gamified cognitive training, and hybrid in-person/digital care models. Representative examples of sub-topic structures for two primary clusters are illustrated in Figure 5.

This fine-grained approach enhanced interpretability and facilitated the identification of overlapping or converging research directions—an essential component for detecting cross-disciplinary integration in rehabilitation science.

### 3.4. Comparison of LDA-Based Topic Modeling with Existing Solutions

SciVal and OpenAlex were selected as external reference systems because they represent widely used but structurally different approaches to large-scale scientometric topic classification. SciVal provides proprietary topic clusters derived from Scopus-indexed literature, whereas OpenAlex offers an open and broader topic structure. The comparison was limited to indicators available across the examined systems, including topic numbers and sizes, corpus coverage, thematic aggregation, and fragmentation. Because the platforms use different publication sets and do not provide comparable underlying model specifications, the analysis was intended to contextualize differences in topic organization rather than to benchmark semantic quality or algorithmic performance.

In SciVal, 1394 topics were identified across the same thematic area, with topic sizes ranging from one to 1092 publications. Of these, only 33 topics (24%) contained ≥200 articles, and 467 topics (40%) contained 20–199 articles. The remaining 36% of articles were scattered across numerous micro-topics with fewer than 20 publications each. This fragmentation limited semantic cohesion and hindered assessment of overarching research priorities. Additionally, SciVal topic names are object–subject-oriented (e.g., “Exoskeletons; Upper Extremity; Stroke Rehabilitation”) and rarely reflect conceptual research goals or outcomes.

OpenAlex, by contrast, provides a broader aggregation of research areas, listing the top 200 topics (231–8728 publications each) for 2022–2025. Although this approach enhances linguistic readability and scope, it lacks transparency regarding corpus coverage and uncategorized articles. Topic labels typically describe either the object or the subject of research rather than the integrative theme.

In comparison, the LDA-based approach presented here produced ten large, semantically cohesive themes that collectively covered 100% of the corpus (Table 1). This framework allows for more precise delineation of thematic boundaries, better reflection of actual research priorities, and easier interpretability for domain experts.

The three approaches were compared using structural indicators that were consistently available across their outputs. These indicators included corpus size, number of topics, topic-size range, proportion of publications represented by the displayed topics, and degree of thematic fragmentation. The LDA framework assigned all 55,711 publications to ten primary topics, ranging from 1511 to 9140 publications. SciVal identified 1394 topics among 57,201 publications, with topic sizes ranging from one to 1092 publications. Only 33 SciVal topics contained at least 200 publications, whereas 467 contained between 20 and 199 publications. The OpenAlex comparison included its 200 highest-ranked topics, ranging from 231 to 8728 publications. However, the available output did not allow the proportion of uncategorized publications or complete corpus coverage to be established with the same precision.

These indicators demonstrate differences in aggregation level, topic-size distribution, and corpus coverage among the three approaches. The LDA-based framework produced a smaller number of broad thematic groups covering the complete study corpus, whereas SciVal and OpenAlex provided more granular topic structures based on different classification procedures and publication sets. This comparison should therefore be interpreted as a structural and descriptive benchmark rather than as a direct quantitative assessment of semantic model quality. Topic coherence, topic diversity, and log perplexity were calculated only for the LDA model because the internal model outputs required to derive equivalent metrics were not available for SciVal and OpenAlex.

## 4. Discussion

The analysis of clinical rehabilitation publications from the early post-pandemic period revealed ten primary research clusters and three overarching trends characterizing the reorganization of the field. These trends reflected the increasing importance of psychosocial and quality-of-life outcomes, technology-supported rehabilitation, and multidisciplinary models of care.

Comparison with SciVal and OpenAlex demonstrated substantial differences in classification granularity, topic-size distribution, corpus coverage, and practical interpretability. Because these systems use different publication sets, classification procedures, and output structures, the comparison was treated as a structural benchmark rather than as a direct test of algorithmic performance. The LDA-based map provided a broad, corpus-specific representation suitable for examining field-level priorities, whereas SciVal and OpenAlex offered more granular classifications developed for different scientometric purposes.

### 4.1. Evolution of the Rehabilitation Paradigm

In recent years, the paradigm of rehabilitation has shifted from a purely medical model focused on compensating for functional losses to a multidisciplinary and holistic approach aimed at restoring comprehensive physical, psychological, and social well-being. This transition aligns with the increasing use of predictive and computational modeling to assess multifactorial determinants of recovery, including physical activity, comorbidities, and psychosocial adaptation.

Within this framework, prognostic modeling serves as a critical tool for identifying high-risk factors and designing individualized rehabilitation trajectories. The growing integration of digital health technologies, including remote monitoring and algorithmic prediction of recovery outcomes, reflects a trend toward data-driven and patient-centered care.

### 4.2. Contextualizing Topic Modeling in Scientometrics

Scientometric topic modeling aims to extract latent structures from large corpora of publications, thereby identifying clusters that represent coherent thematic directions. Among the various methods developed for this purpose, Latent Dirichlet Allocation (LDA) remains one of the most established probabilistic approaches for inferring hidden semantic relationships within textual data.

Alternative approaches have emerged, each with distinct advantages and limitations:

- Word Embeddings [31] transform words into numerical vectors that encode contextual similarity, providing the foundation for many modern NLP models but lacking explicit topic-level interpretability;
- Latent Semantic Analysis (LSA) [32] captures conceptual relations via matrix decomposition but disregards probabilistic word-topic relationships;
- Non-Negative Matrix Factorization (NMF) [33] offers non-negative decomposition for better interpretability but may underperform in complex, heterogeneous datasets;
- Machine Learning models (e.g., BERT) [34] utilize deep contextual embeddings for semantic representation; however, they require substantial computational resources and large-scale fine-tuning;
- Additive Regularization of Topic Models (ARTM) [34,35] extends probabilistic frameworks with regularization, improving topic separation and integration of metadata;
- Deep Learning approaches [36] capture complex nonlinear dependencies but often lack transparency and reproducibility in thematic mapping;
- Graph-based models [37] represent relationships among words or topics as network structures but require extensive preprocessing and cannot independently yield interpretable thematic categories.

Although these techniques have advanced substantially, most remain experimental and demand extensive manual post-processing to achieve coherent, human-interpretable results. Consequently, large bibliometric systems continue to rely on validated but simplified clustering algorithms that trade interpretability for scalability.

### 4.3. Limitations of Existing Scientometric Systems

Commercial and open-source topic-modeling solutions, such as SciVal and OpenAlex, benefit from large-scale validation and reproducibility; however, they exhibit critical limitations compared with more flexible probabilistic or neural models.

Most notably, these systems:

- Lack explicit representations of inter-topic relationships, which limits understanding of interdisciplinary dynamics [38].
- Employ coarse-grained classification schemes that obscure nuanced thematic developments, particularly in cross-disciplinary domains [39].
- Struggle with semantic sparsity, short-text irregularity, and instability due to random initialization [40,41,42].
- Produce topic clusters that may diverge from expert intuition, hindering interpretability and user trust [43].
- Such limitations underscore the need for approaches that balance computational scalability with semantic precision.

### 4.4. Addressing Key Challenges Through Combined Human–Machine Modeling

The methodological contribution of the present study lies in the analytical framework rather than in the development of a new topic-modeling algorithm. LDA remains an established and computationally accessible probabilistic method, but its bag-of-words topic representations often require additional interpretation, particularly when short or domain-specific texts are analyzed. Recent approaches based on generative labeling and time-sensitive analysis of scientific corpora have sought to improve the interpretability and specificity of topic representations [44,45]. At the same time, coherence, topic diversity, and log perplexity assess different statistical properties of a model and cannot individually establish its semantic validity or practical usefulness. Recent evaluation frameworks therefore recommend combining automated metrics with purpose-oriented semantic assessment and human interpretation [46].

The novelty of this work should therefore be interpreted at the level of study design and domain application. Previous LDA-based analyses in rehabilitation have generally focused on individual technologies or narrowly defined patient groups [47,48]. In contrast, the present analysis examines clinical rehabilitation as an interdisciplinary field, uses the early post-pandemic period to characterize paradigm-level changes, resolves broad thematic domains into more specific subtopics, and directly compares the resulting structure with two external scientometric systems. This combined perspective enables assessment not only of which topics are represented, but also of how alternative classification approaches define the scale, boundaries, and internal organization of the field.

Accordingly, the framework combined corpus-level LDA modeling with secondary modeling within each primary cluster. It also incorporated complementary quantitative evaluation, transformer-generated provisional labels, independent expert refinement, and comparison with external scientometric classification systems. Secondary modeling produced a practical two-level structure. Broad rehabilitation domains could therefore be subdivided into more specific research directions. Recent expert-in-the-loop hierarchical approaches similarly show that selective partitioning of heterogeneous topics can reveal levels of thematic granularity that remain obscured in flat topic models [49]. Generative language models were used only to produce provisional descriptive labels. Final interpretation remained under expert control because natural-language labels may improve readability but do not independently validate the underlying topic structure [44,46]. The comparison with SciVal and OpenAlex was therefore treated as a structural and coverage-based benchmark rather than as evidence of the general algorithmic superiority of LDA.

The thematic structure presented in Figure 4 indicates that the transformation of rehabilitation research is occurring primarily through the integration of new methods and outcome priorities within established clinical domains rather than through their replacement. Condition-specific clusters—including stroke, orthopedic, cardiopulmonary, oncological, pediatric, and traumatic brain injury rehabilitation—are connected by recurring themes of technology-assisted treatment, functional recovery, quality of life, and multidisciplinary care. Within these clusters, virtual reality, robotics, stimulation technologies, exercise-based interventions, and patient-reported outcomes emerge as cross-cutting components rather than isolated research niches. The expanding role of rehabilitation robotics and allied digital technologies supports this interpretation while also emphasizing the need for clinically oriented implementation and evaluation [50]. Evidence across cancer, heart failure, and respiratory disease likewise demonstrates the growing relevance of multidisciplinary rehabilitation to quality-of-life outcomes [51]. In parallel, recent rehabilitation research identifies systematic collection of patient-reported outcome measures as an essential component of evaluating patients’ functional status and perceived treatment benefits [52]. In the present study, we attempted to mitigate these issues by integrating automated topic extraction with expert manual validation, combining the efficiency of LDA-based modeling with the interpretive rigor of human expertise. This hybrid strategy enhanced thematic coherence and ensured semantic alignment with domain knowledge.

Through this approach, we identified three dominant trends shaping current rehabilitation research:

- Quality of life and psychosocial recovery:

Post-COVID-19 rehabilitation increasingly emphasizes psychosocial well-being, incorporating comprehensive psychological evaluation, self-monitoring, social reintegration, and gamified behavioral interventions to enhance motivation and adherence [53,54,55,56,57].

- Technological integration:

The expansion of telemedicine, wearable sensors, and virtual reality technologies is transforming rehabilitation into a continuously monitored, adaptive process [58,59,60]. Such technologies bridge physical and digital therapy environments, enabling real-time feedback and individualized treatment.

- Comprehensive multidisciplinary rehabilitation:

To connect the model-derived topic structure more directly with clinical rehabilitation research, Supplementary Table S1 presents two representative publications from the top 10% most-cited subset for each of the ten primary topics [61,62,63,64,65,66,67,68,69,70,71,72,73,74,75,76,77,78,79,80].

The transition toward integrative rehabilitation underscores collaboration between medical, psychological, and social disciplines to maximize functionality and long-term recovery [81,82,83]. Standardization of protocols and the adaptation of international best practices further support this transition.

### 4.5. Limitations of the Current Study

Several limitations should be considered. First, the analysis used a single LDA configuration without sensitivity testing or direct comparison with alternative topic-modeling methods; therefore, the exact number, boundaries, and sizes of the topics may depend on the selected algorithm and hyperparameters. The resulting structure should be regarded as one interpretable representation of the corpus rather than a definitive taxonomy of rehabilitation research. Second, expert-assisted exclusion of non-clinical records and verification of topic labels may have introduced subjectivity, particularly for ambiguous publications and smaller, semantically heterogeneous topics. Consensus procedures reduced this risk, although its magnitude could not be quantified because formal inter-rater agreement was not assessed. Third, comparison with SciVal and OpenAlex was limited to available structural indicators, including topic counts, size distributions, corpus coverage, and fragmentation. Differences in publication sets and the absence of underlying model specifications precluded direct comparison of semantic quality or algorithmic performance. These limitations restrict the precision and generalizability of the findings but do not invalidate their exploratory description of the broad thematic structure of the analyzed corpus. The findings should not be interpreted as evidence of a unique or optimal classification, general methodological superiority, or the clinical effectiveness and implementation of the identified research directions.

### 4.6. Implications for Future Scientometric Research

The methodological framework developed here illustrates the potential of LDA-based topic modeling for capturing emerging research trends in rehabilitation and beyond. By incorporating continuous corpus updates, coherence-based optimization, and hybrid human–machine interpretation, such models can serve as dynamic observatories for mapping scientific evolution across disciplines.

Future developments may include contextualized probabilistic models (e.g., BERTopic or neural LDA variants) capable of incorporating citation networks, metadata, and temp oral information, thus providing a more multidimensional view of scientific progress.

## 5. Conclusions

The early post-pandemic rehabilitation literature reflects a growing convergence of research on physical recovery, quality of life, psychosocial adaptation, social participation, and long-term self-management. Digital and assistive approaches, including telerehabilitation, wearable monitoring, virtual reality, and robot-assisted therapy, are increasingly investigated as means of extending rehabilitation beyond conventional face-to-face sessions. These directions indicate that the research agenda is shifting from isolated, diagnosis-specific interventions toward more continuous, multidisciplinary, and patient-centered models of rehabilitation.

For health policy-makers, this thematic landscape may inform priority setting related to equitable digital rehabilitation infrastructure, professional training, interoperable systems, shared outcome standards, and implementation research involving diverse patient populations. For medical specialists, it highlights areas in which multidimensional assessment, interdisciplinary coordination, and carefully selected digital or assistive technologies may support individualized rehabilitation pathways. These implications represent priorities emerging from the publication landscape and should not be interpreted as evidence of the comparative clinical effectiveness of particular interventions. Decisions concerning clinical implementation require prospective evaluation of safety, effectiveness, accessibility, and long-term patient outcomes.

## Figures and Tables

**Table 1:** Comparison of topics based on three approaches: 10 topic groups formulated using LDA modeling on 55,711 publications (includes 100% articles), top 20 topics from 57,201 SciVal publications (includes 19% articles), and top 20 topics from 247,100 OpenAlex publications (includes about 20% of articles).

Name by LDA Topic Modeling (N of Articles)	Most Relevant SciVal Topics (N of Articles)	Most Relevant OpenAlex Topics
COVID-19 and Rehabilitation Care: Health, Quality of Life, and the Physical Experience in Qualitative Analyses (5390) Integrating Cancer and Cardiac Rehabilitation: Evaluating Exercise, Surgery, and Cardiovascular Treatments (9140) Knee Rehabilitation with Exoskeleton and Robot-Assisted Therapy: Enhancing Control and Functionality Post-Arthroplasty (6080) Traumatic Brain Injury and COVID-19 Cohort Rehabilitation: The Role of Virtual Reality in Syndrome and Disorder Treatment (2230) Stroke Rehabilitation Enhanced by Gait and Limb Stimulation: Functional Training Effects on Upper Limb and Walking Capabilities (5387) Childhood Disability and Spinal Cord Injury Rehabilitation: Advances in Research and Developmental Assessment (5894) Evaluating Rehabilitation Protocols: A Meta-Analysis of Randomized Controlled Trials, Exercise Interventions, and Pain Management (8461) Chronic Pulmonary Rehabilitation: Managing Heart Failure and Obstructive Lung Diseases Through Effective Treatments (1511) Muscle Strength Recovery in Anterior Cruciate Ligament Reconstruction: Rehabilitation and Sports Implant Efficacy (7404) Fracture Management and Rehabilitation: Surgical Treatments and Outcomes in Lumbar and Cervical Repairs (4214)	Exoskeletons; Upper Extremity; Stroke Rehabilitation (1092) Exoskeletons; Robotics; Foot Orthoses (1042) Telerehabilitation; Virtual Reality; Upper Extremity (926) Gait; Stroke Rehabilitation; Exercise (816) Chronic Obstructive Lung Disease; Pulmonary Rehabilitation; Forced Expiratory Volume (782) Upper Extremity; Stroke Rehabilitation; Arm (668) Anterior Cruciate Ligament Reconstruction; Knee; Return to Sport (586) Telerehabilitation; COVID-19; E-Health (477) Postoperative Complications; Enhanced Recovery; Colorectal Surgery (464) Accidental Falls; Parkinson’s Disease; Dual Task (431) Radiological Findings; Clinical Features; COVID-19 (372) Childhood Cancer Survivor; Strength Training; Exercise (361) Multiple Sclerosis; Gait; Walking (353) Intensive Care Units; Critical Illness; Mobilization (349) Deglutition Disorders; Swallowing; Upper Esophagus Sphincter (322) Outcome; Community Integration; Return to Work (305) Motor Imagery; Brain Computer Interface; Electroencephalogram (305) Electromyography; Artificial Limbs; Muscle (293) Cerebral Palsy; Spasticity; Preschool Children (291) Outcome; Geriatrics; Femur Neck (289)	Principles and Interventions in Stroke Rehabilitation (8728) Neurological Manifestations of COVID-19 Infection (4015) Cardiac Rehabilitation and Cardiovascular Health Interventions (3121) Global Burden of Chronic Pain (2903) Dental Implantology and Peri-implant Diseases (2734) Classification and Interventions for Cerebral Palsy (2536) Standardisation and Management of COPD (2455) Anterior Cruciate Ligament Injuries in Athletes (2441) Analysis of Electromyography Signal Processing (2348) Shoulder Pathology and Treatment Outcomes (2238) Brain–Computer Interfaces in Neuroscience and Medicine (2063) Lower Limb Exoskeleton Robotics (1964) Epidemiology and Impact of Traumatic Brain Injury (1936) Pathophysiology and Treatment of Spinal Cord Injury (1745) Epidemiology of Sports-Related Musculoskeletal Injuries (1619) Projections and Outcomes of Arthroplasty Surgery in the US (1457) Cancer Survivorship and Quality of Life (1433) Epidemiology and Prevention of Cardiovascular Diseases in Russia (1325) Incidence and Management of Hip Fractures (1311) Treatment and Outcomes of Hand Injuries (1284)

## Data Availability

The original contributions presented in this study are included in the article. Further inquiries can be directed to the corresponding author.

## Supplementary Materials

The following supporting information can be downloaded at: https://www.mdpi.com/article/10.3390/healthcare14162661/s1, Table S1: Representative highly cited publications illustrating the ten LDA-derived topics in clinical rehabilitation.

## Abbreviations

The following abbreviations are used in this manuscript:

LDA	Latent Dirichlet Allocation
