# Supplementary material for: Thematic Structure of Clinical Rehabilitation Research in the Early Post-Pandemic Period: An LDA-Based Analysis of Publications from 2022 to 2025
Source: Healthcare (Basel). 2026 Aug 21;14(16):2661. doi: 10.3390/healthcare14162661 (PMC13511976; doi:10.3390/healthcare14162661)
Supplement: Supplementary file 1 [file healthcare-14-02661-s001.zip › healthcare-4418091-supplementary.pdf]

Table S1. Representative highly cited publications illustrating the ten LDA-derived topics in clinical rehabilitation.

LDA-derived topic	Representative article	DOI
1. COVID-19 and Rehabilitation Care: Health, Quality of Life, and the Physical Experience in Qualitative Analyses	Symptoms and management of long COVID: A scoping review (2024) [61]	https://doi.org/10.1111/jocn.16150
	A systematic review on physical function, activities of daily living and health-related quality of life in COVID-19 survivors (2023) [62]	https://doi.org/10.1177/17423953221089309
2. Integrating Cancer and Cardiac Rehabilitation: Evaluating Exercise, Surgery, and Cardiovascular Treatments	Effect of Multimodal Prehabilitation on Reducing Postoperative Complications and Enhancing Functional Capacity Following Colorectal Cancer Surgery: The PREHAB Randomized Clinical Trial (2023) [63]	https://doi.org/10.1001/jamasurg.2023.0198
	Exercise-based cardiac rehabilitation for coronary heart disease: a meta-analysis (2023) [64]	https://doi.org/10.1093/eurheartj/ehac747
3. Knee Rehabilitation with Exoskeleton and Robot-Assisted Therapy: Enhancing Control and Functionality Post-Arthroplasty	Rehabilitation of Total Knee Arthroplasty by Integrating Conjoint Isometric Myodynamia and Real-Time Rotation Sensing System (2022) [65]	https://doi.org/10.1002/advs.202105219
	Adaptive Synergetic Motion Control for Wearable Knee-Assistive System: A Rehabilitation of Disabled Patients (2022) [66]	https://doi.org/10.3390/act11070176
4. Traumatic Brain Injury and COVID-19 Cohort Rehabilitation: The Role of Virtual Reality in Syndrome and Disorder Treatment	INCOG 2.0 Guidelines for Cognitive Rehabilitation Following Traumatic Brain Injury, Part IV: Cognitive-Communication and Social Cognition Disorders (2023) [67]	https://doi.org/10.1097/HTR.0000000000000835
	Effects of home-based virtual reality telerehabilitation system in people with multiple sclerosis: A randomized controlled trial (2024) [68]	https://doi.org/10.1177/1357633X211054839
5. Stroke Rehabilitation Enhanced by Gait and Limb Stimulation: Functional Training Effects on Upper Limb and Walking Capabilities	Efficacy of robotic exoskeleton for gait rehabilitation in patients with subacute stroke: a systematic review (2022) [69]	https://doi.org/10.23736/S1973-9087.21.06846-5
	Action observation training and brain-computer interface controlled functional electrical stimulation enhance upper extremity performance and cortical activation in patients with stroke: a randomized controlled trial (2022) [70]	https://doi.org/10.1080/09593985.2020.1831114
6. Childhood Disability and Spinal Cord Injury Rehabilitation: Advances in Research and Developmental Assessment	Towards a paradigm shift in pediatric rehabilitation: accelerating the uptake of evidence on participation into routine clinical practice (2022) [71]	https://doi.org/10.1080/09638288.2021.1903102
	Walking naturally after spinal cord injury using a brain–spine interface (2023) [72]	https://doi.org/10.1038/s41586-023-06094-5
7. Evaluating Rehabilitation Protocols: A Meta-Analysis of Randomized Controlled Trials, Exercise Interventions, and Pain Management	Efficacy of rehabilitation on reducing pain in muscle-related temporomandibular disorders: A systematic review and meta-analysis of randomized controlled trials (2022) [73]	https://doi.org/10.3233/BMR-210236

	Exercise Therapy Is Effective for Improvement in Range of Motion, Function, and Pain in Patients With Frozen Shoulder: A Systematic Review and Meta-analysis (2022) [74]	https://doi.org/10.1016/j.apmr.2021.07.806
8. Chronic Pulmonary Rehabilitation: Managing Heart Failure and Obstructive Lung Diseases Through Effective Treatments	Using a smartphone application maintains physical activity following pulmonary rehabilitation in patients with COPD: a randomised controlled trial (2023) [75]	https://doi.org/10.1136/thoraxjnl-2021-218338
	Effects of exercise-based cardiac rehabilitation delivery modes on exercise capacity and health-related quality of life in heart failure: a systematic review and network meta-analysis (2022) [76]	https://doi.org/10.1136/openhrt-2021-001949
9. Muscle Strength Recovery in Anterior Cruciate Ligament Reconstruction: Rehabilitation and Sports Implant Efficacy	Aspetar clinical practice guideline on rehabilitation after anterior cruciate ligament reconstruction (2023) [77]	https://doi.org/10.1136/bjsports-2022-106158
	ACL Reconstruction Rehabilitation: Clinical Data, Biologic Healing, and Criterion-Based Milestones to Inform a Return-to-Sport Guideline (2022) [78]	https://doi.org/10.1177/19417381211056873
10. Fracture Management and Rehabilitation: Surgical Treatments and Outcomes in Lumbar and Cervical Repairs	The global approach to rehabilitation following an osteoporotic fragility fracture: A review of the rehabilitation working group of the International Osteoporosis Foundation committee of scientific advisors (2022) [79]	https://doi.org/10.1007/s00198-021-06240-7
	Effects of home-based occupational therapy telerehabilitation via smartphone for outpatients after hip fracture surgery: A feasibility randomised controlled study (2022) [80]	https://doi.org/10.1177/1357633X20932434
